# LncRNA and mRNA expression profiles reveal the potential roles of lncRNA contributing to regulating dural penetration in clival chordoma

**DOI:** 10.18632/aging.103294

**Published:** 2020-06-13

**Authors:** Jiwei Bai, Yixuan Zhai, Shuai Wang, Mingxuan Li, Shuheng Zhang, Chuzhong Li, Songbai Gui, Qi Li, Yazhuo Zhang

**Affiliations:** 1 Beijing Neurosurgical Institute, Capital Medical University, Beijing 100070, China; 2 Department of Neurosurgery, Beijing Tiantan Hospital, Capital Medical University, Beijing 100070, China; 3 China National Clinical Research Center for Neurological Diseases, Beijing Tiantan Hospital, Capital Medical University, Beijing 100070, China; 4 Department of Neurosurgery, First Affiliated Hospital, Zhengzhou University, Zhengzhou 450052, China; 5 Department of Neurosurgery, Anshan Central Hospital, Anshan 114001, China

**Keywords:** clival chordomas, dural penetration, LncRNAs, microarray analysis, biological functions

## Abstract

Chordoma is a rare bone cancer originating from embryologic notochordal remnants. Clival chordomas show different dural penetration ability, with serious dural penetration exhibiting poorer prognosis. The molecular mechanism of dural penetration is not clear. We analyzed lncRNA and mRNA profiles in 12 chordoma patients with different degrees of dural penetration using expression microarrays. The differentially expressed lncRNAs and mRNAs were used to construct a lncRNA-mRNA co-expression network. LncRNAs were classified into lincRNA, enhancer-like lncRNA, or antisense lncRNA. Biological functions for lncRNAs were predicted according to the lncRNA-mRNA network and adjacent coding genes by pathway analysis. The 2760 lncRNAs and 3988 mRNAs were differentially expressed in chordomas between two groups of patients with and without dural penetration. Possible pathway involvement of the significance among the 55 lncRNAs located in the lncRNA-mRNA network, 24 lincRNAs, 7 enhancer-like lncRNAs, and 14 antisense lncRNAs include cell adhesion, metastasis, invasion, proliferation, and apoptosis. Expression of 10 lncRNAs and mRNAs, and epidermal growth factor mRNA with two identified lncRNAs were subsequently verified by qRT-PCR in chordoma tissues. Our report predicts the biological functions of many lncRNAs which may be used as diagnostic and prognostic biomarkers as well as therapeutic targets during the process of dural penetration in chordoma.

## INTRODUCTION

Chordomas are rare, malignant, primary bone tumors, with an incidence of approximately 0.1/100,000/year [1]. They originate from remnants of the embryologic notochord, with the most common sites being the sacrum (50-60%), clivus region of the skull base (25-35%), and cervical and thoracolumbar vertebrae (15%) [2]. The standard treatment for chordoma is maximum surgical resection. However, local recurrence and metastasis can occur after surgical resection, and current radiotherapy and chemotherapeutic protocols are largely ineffective [3–5]. Advances in targeted molecular therapy may offer new treatment options for chordoma patients [6–9].

Chordomas are characterized as being aggressive and locally invasive, and clivus chordomas have different degrees of dural penetration. Some tumors severely penetrate the dura mater, with little bone invasion, while others show little or no dura mater penetration with extensive skull base bone invasion. Our previous study showed chordoma patients with serious dural penetration have poorer prognosis and patients without dural penetration have longer overall survival and progressive-free survival [10].

Long non-coding RNAs (lncRNAs) are greater than 200 nucleotides in length and regulate gene expression at transcriptional and post-transcriptional levels [11, 12]. Transcriptional patterns and locations in the genome of lncRNAs are complex. Based on their locations relative to protein-coding genes, lncRNAs are roughly classified as intergenic (between genes), intragenic/intronic (within genes), and antisense [13]. Large intergenic non-coding RNAs (lincRNAs) are emerging as key regulators of diverse cellular processes as previously identified [14]. Enhancer-like lncRNAs are also intergenic lncRNAs and locate in the enhancer region of the genome, and which can activate the transcription of adjacent genes [15]. Antisense lncRNAs have been shown to regulate the corresponding sense coding gene at the transcriptional or post-transcriptional level, which can exert biological functions [16].

LncRNAs participate in various aspects of cell biology, including cell proliferation, apoptosis, differentiation, invasion and metastasis, and are commonly dysregulated in multiple types of cancers [17]. Recent research related to chordoma demonstrated that differential regulation of lncRNA expression correlated with the expression changes in protein coding genes, which indicates a comprehensive lncRNA-coding gene co-expression network. For example, one study revealed that lncRNA MEG3 contributes to the pathogenesis of chordoma development by regulating imprinted gene DLK1 [18]. Moreover, lncRNA LOC554202 may modulate chordoma cell proliferation and invasion by recruiting EZH2 and regulating miR-31 expression [19]. However, the functional significance of lncRNAs to dural penetration of chordoma is not yet clear.

In the present study, we identified differentially expressed lncRNAs and gene expression profiles of chordomas with varying degrees of dural penetration and constructed lncRNA-gene co-expression networks to reveal the functional role of lncRNAs in regulating dural penetration of clivus chordoma. Among the top differentially expressed lncRNAs, we identified lincRNA, antisense lncRNA, and enhancer lncRNA which may contribute to the dural penetration biological processes in chordoma.

## RESULTS

### Patient characteristics

**Figure 1:**
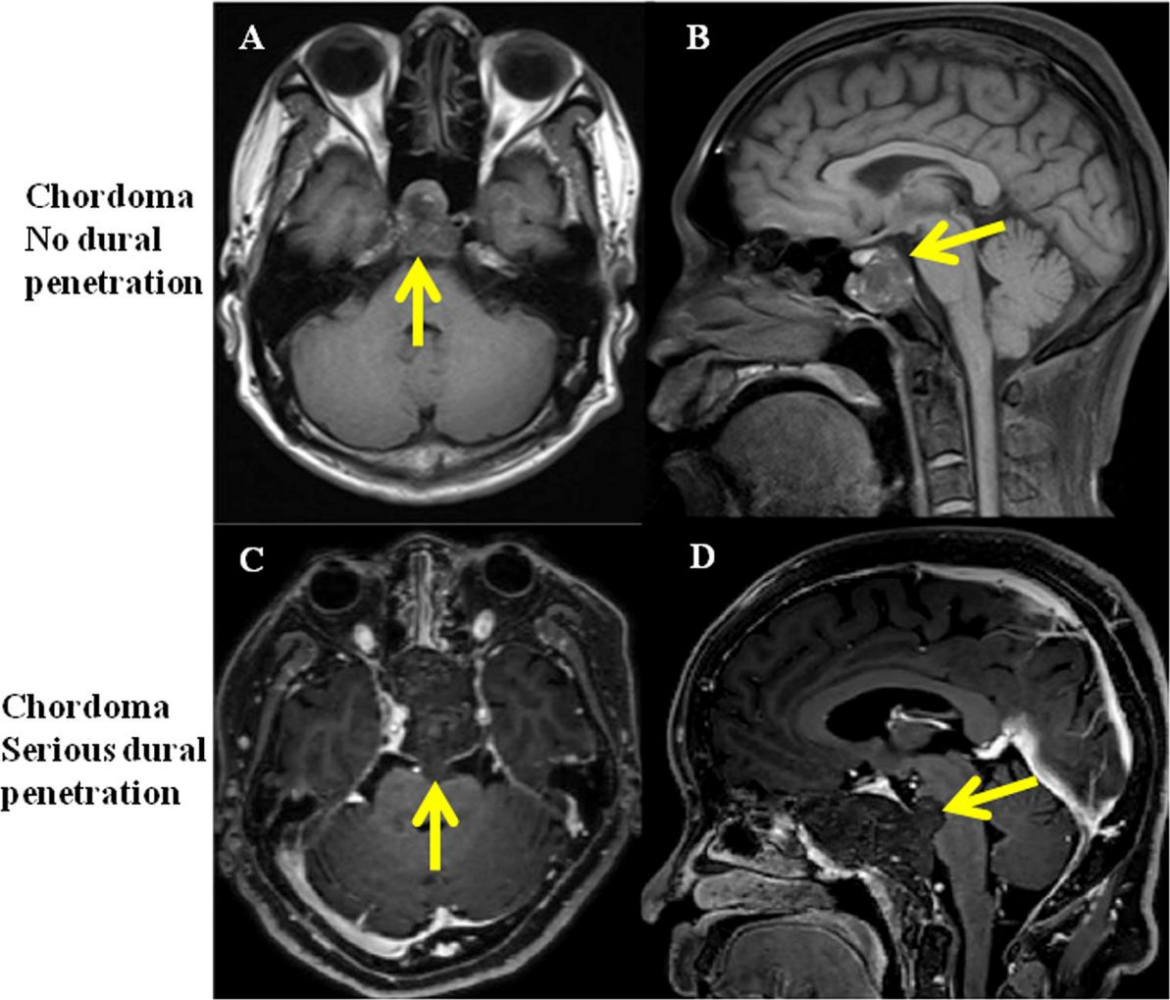
**MRI of clival chordoma patients with no dural penetration and with serious dural penetration.** (**A**, **B**) Same chordoma patient with no dural penetration, yellow arrows show the integrity of dura mater; (**C**, **D**) Same chordoma patient with serious dural penetration, yellow arrows show tumor penetrate the dura mater and invade into intracranial space.

**Figure 2:**
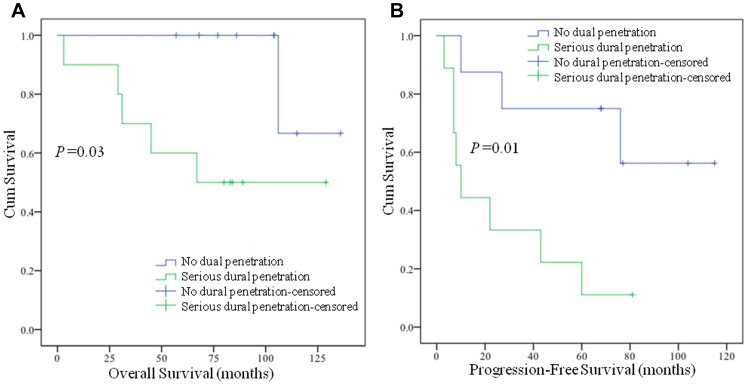
**Kaplan-Meier survival analysis of chordoma patients.** (**A**) Chordoma patients with serious dural penetration have shorter overall survival time than patients with no dural penetration (*P* = 0.03); (**B**) Chordoma patients with serious dural penetration have shorter progression-free survival time than patients with no dural penetration (*P* = 0.01).

Twenty patients were enrolled in the study and were equally classified into no dural penetration group and serious dural penetration group. The tumor with no dural penetration demonstrated expansive growth, like a “bubble” in magnetic resonance imaging (MRI) and dural perforation was not found in surgical procedure (Figure 1A, 1B). In the chordomas samples with serious dural penetration, a bulge from the tumor into intracranial areas could be found in MRI, whereas dural perforation was found during the operation (Figure 1C, 1D). The patients’ demographic features were summarized in Table 1. The differences in age, sex and length of follow-up from surgery were not statistically significant between the two groups. However, the overall survival time and progression-free time were significantly differential, with poorer outcomes seen in the group with serious dural penetration (Figure 2A, 2B).

**Table 1:** Patients’ demographic features, survival and recurrence information.

	**No dural penetration**	**Serious dural penetration**	**P-value**
Sex (Male vs. Female)	40% vs. 60%	50% vs. 50%	0.65
Age (mean ± SD)	46.6 ± 17.0	41.0 ± 17.9	0.48
Follow-up period (months) (mean ± SD)	92.1 ± 24.9	64.0 ± 36.9	0.06
Overall survival (months) (mean, 95%CI)	126.0, (110.0,142.0)	82.0, (51.5, 112.5)	0.03
Progression-free survival (months) (mean, 95%CI)	83.6, (54.6, 112.6)	26.8 (9.5, 44.1)	0.01

### Distinctive lncRNA and mRNA expression between no dural penetration and serious dural penetration of clival chordoma tissues

**Figure 3:**
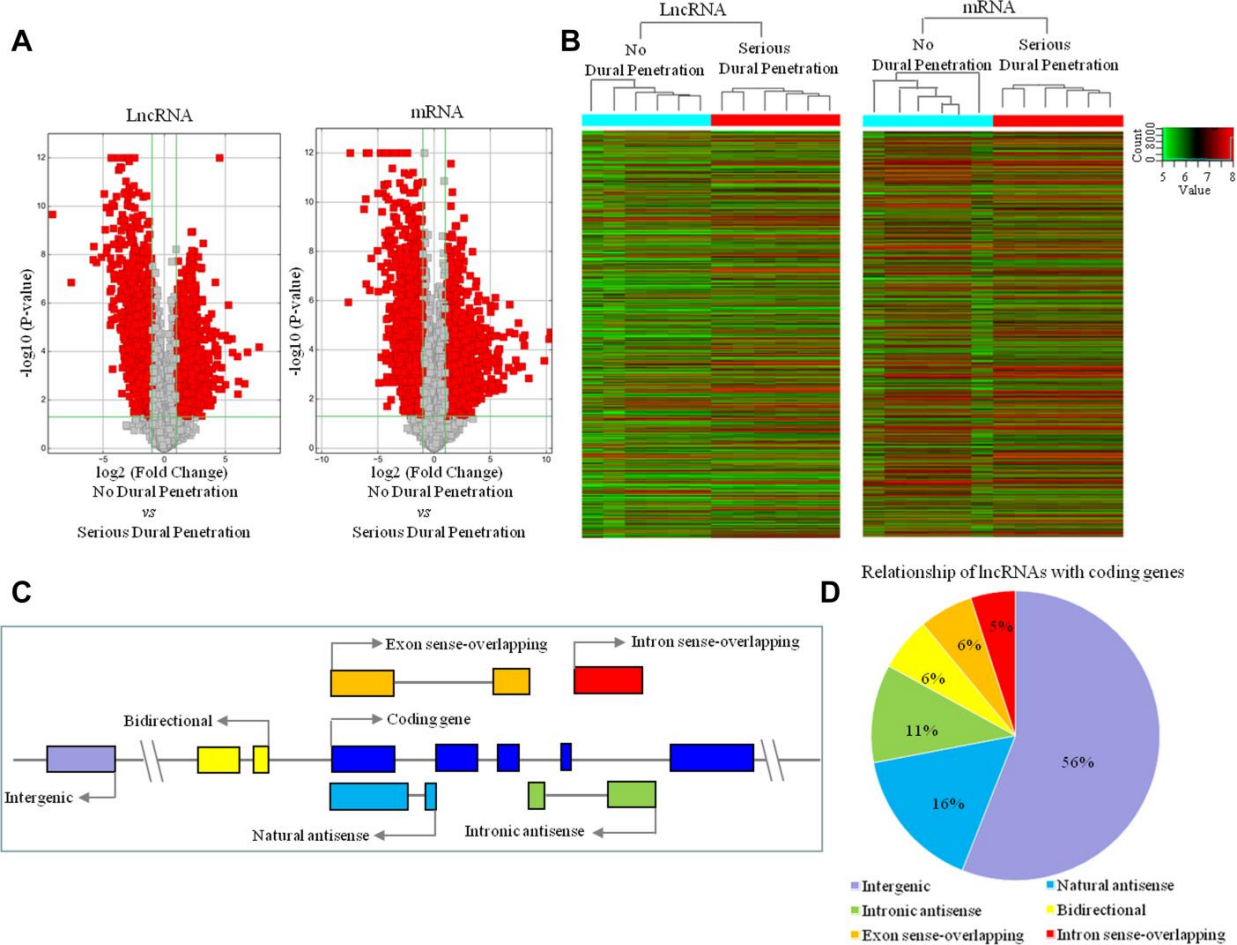
**LncRNA and mRNA expression profile changes between serious dural penetration and no-dural penetration in chordoma.** (**A**) Volcano plot analysis of 2760 lncRNAs and 3988 mRNAs that are differentially expressed between no dural penetration group and serious dural penetration group; and red boxes represent ≥ 2-fold change difference and *P* ≤ 0.05. (**B**) Hierarchical clustering of all samples revealed the nonrandom partitioning of samples into two major groups: one group containing six no dural penetration samples and another group containing six serious dural penetration samples. Each column represents one sample and each row represents one lncRNA or mRNA probe set. (**C**) Transcriptional patterns of lncRNAs. Nature antisense: the lncRNA is transcribed from the antisense strand and overlapping with a coding transcript; Intronic antisense: the lncRNA is transcribed from the antisense strand without sharing overlapping exons; Intron sense-overlapping: the lncRNA is overlapping the intron of a coding transcript on the same genomic strand; Exon sense-overlapping: the lncRNA is overlapping the exon of a coding transcript on the same genomic strand; Intergenic: there are no overlapping or bidirectional coding genes nearby the lncRNA; Bidirectional: the lncRNA is oriented head to head to a coding gene within 1000 bp. Arrow represent transcription direction. (**D**) Subgroup analysis of altered lncRNAs in relation to their nearby coding genes.

We compared the lncRNA and mRNA expression patterns in the tissue samples between clival chordoma patients with no dural penetration (n = 6) and with serious dural penetration (n = 6). The 2760 lncRNAs and 3988 mRNAs were identified that had significantly differential expression in no dural penetration samples compared to serious dural penetration samples (fold change ≥ 2, *P* < 0.05, FDR < 0.05; Figure 3A; Supplementary Tables 1, 2). Of these 2760 lncRNAs, 1773 were down-regulated while the rest 987 were up-regulated. Such differentiation signified their potential roles in dural penetration. The Hierarchical cluster analysis showed systematic variations in the expression of differential lncRNAs and mRNAs in the no dural penetration and serious dural penetration samples (Figure 3B). Thus, the lncRNA and mRNA expression signatures identified here were likely representative.

To identify the characteristics of 2760 differentially expressed lncRNAs, we analyzed the genomic location of these lncRNAs. The gene loci of lncRNAs were found either between two coding genes (intergenic), overlapping exons or intron in sense or antisense orientation, and bidirectional (Figure 3C). Our results indicated that 56% differentially expressed lncRNAs were intergenic, 6% were bidirectional and the remaining were associated with known coding genes in a sense (11%) or antisense (27%) configuration (Figure 3D).

### Construction of lncRNA-mRNA co-expression network and prediction of lncRNA diverse functional roles based on this network

**Figure 4:**
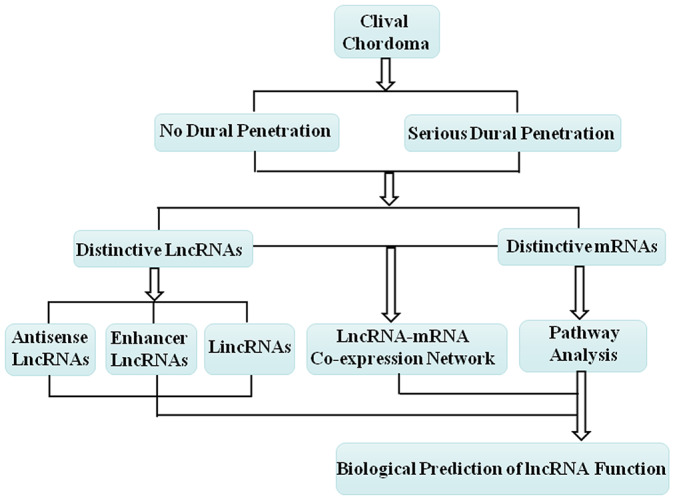
**Schematic overview of the workflow for construction of the lncRNA-mRNA co-expression network and prediction of the lncRNA biological function.**

The lncRNA-mRNA co-expression network was constructed following the workflow shown in Figure 4. Based on the expression profiles of the differentially expressed lncRNAs and mRNAs between the no dural penetration and serious dural penetration chordoma samples, we constructed lncRNA-mRNA co-expression network (Supplementary Figure 1). All of the mRNAs and lncRNAs involved in the networks were shown in Supplementary Table 3.

The identification of target genes is important for exploring the molecular mechanisms underlying lncRNAs function. The KEGG pathway analysis was applied to analyze the significant pathway of the altered 3988 mRNAs. The pathway analysis revealed that there are 62 and 38 significant pathways corresponding to the up and down-regulated mRNAs respectively (*P* < 0.05, Supplementary Tables 4, 5). These significant pathways contained focal adhesion, PI3K-AKT signaling pathway, cell adhesion molecules (CAMs), apoptosis, MAPK signaling, etc, which play key roles in various biological processes of dural penetration in chordoma.

**Figure 5:**
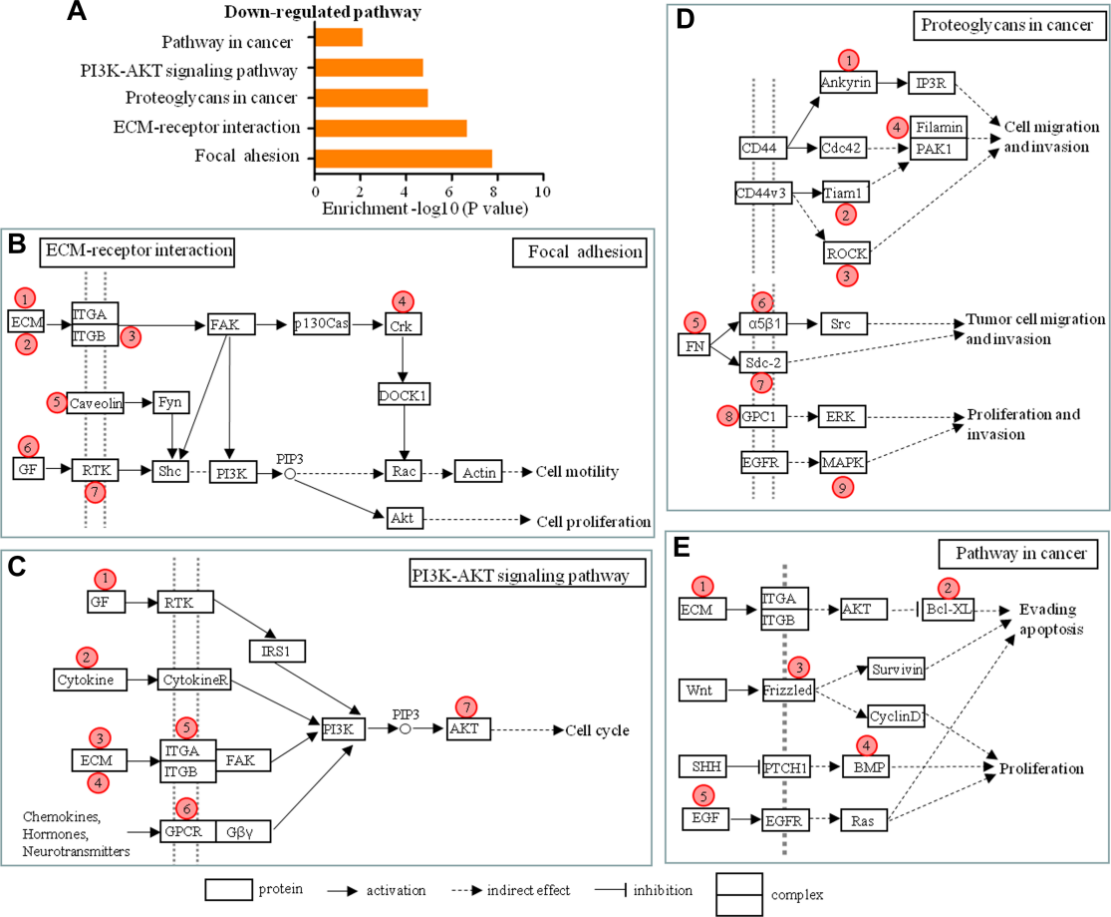
**Schematic overviews of possible signaling pathways in which the 55 lncRNAs in the lncRNA-mRNA co-expression network participated.** (**A**) The pathway analysis applied for 28 mRNAs that were aberrantly expressed in different degree of dural penetration of chordoma and co-expressed with 55 lncRNAs showed 5 down-regulated pathways (*P* < 0.05). (**B**–**E**) Schematic overviews of the signaling pathways in which the 55 lncRNAs in the lncRNA-mRNA co-expression network probably participated. Red circles represent the location of 28 mRNAs predicted from co-expression network. Detailed descriptions of the lncRNAs and mRNAs are presented in Tables 1–4.

According to the lncRNA-mRNA co-expression network and pathway analysis five down-regulated pathways were identified, and they contributed to various cellular processes, including cell adhesion, migration, invasion, apoptosis and proliferation, and affected the dural penetration ability of chordoma cells (Figure 5A). The 27 mRNAs co-expressed with 55 lncRNAs were involved in these five down-regulated pathways. Therefore, we predicted that those 55 lncRNAs were involved in dural penetration of chordoma. The co-expression status of 55 lncRNAs and 27 mRNAs were shown in the focal adhesion and ECM-receptor interaction pathway (Figure 5B); the PI3K-AKT signaling pathway (Figure 5C); the proteoglycans in cancer pathway (Figure 5D); the pathway in cancer (Figure 5E). Detailed descriptions of the 55 lncRNAs, 27 mRNAs, and five pathways in which they participated were listed in Tables 2–5.

**Table 2:** Detailed descriptions of the lncRNAs and mRNAs in the ECM-receptor interaction and focal adhesion pathway.

Number*	**Protein**	**mRNA-lncRNA**
①	ECM	IBSP ↔ BC028842, XLOC_011172, AC002454.1, RP11-736K20.5, XLOC_011178, XLOC_000424
②	ECM	SPP1 ↔ RP11-469A15.2
③	ITGB	ITGB8 ↔ ANKRD30BP2, RP11-1C1.7
④	Crk	CRK ↔ WDFY3-AS2, AC019068.2
⑤	Caveolin	CAV1 ↔ PSORS1C3
⑥	GF	VEGFA ↔ AC005013.5
⑦	RTK	KDR ↔ XLOC_001338, AC005162.3

* Represents the number in Figure 5B.

**Table 3:** Detailed descriptions of the lncRNAs and mRNAs in the PIK3-AKT signaling pathway.

Number*	**Protein**	**mRNA-lncRNA**
①	GF	EFNA1 ↔ AX747547
②	Cytokine	PRL ↔ U47924.27, EFCAB1, RP11-438D8.2, XLOC_006928, CTD-2501M5.1, XLOC_004244, RP11-308B16.2, RP11-400N13.1, AC007682.1, RP11-254K3.1
③	ECM	COL1A1 ↔ LINC00525
④	ECM	COL6A3 ↔ AC004540.4
⑤	ITGA	ITGA3 ↔ CTC-367J11.1, LOC441204, WDFY3-AS2, LINC00475, CSPG4P5
⑥	GPCR	CHRM2 ↔ IPW
⑦	AKT	AKT3 ↔ XLOC_010305, NOP14-AS1, RP11-72I8.1, KRT18P55

* Represents the number in Figure 5C.

**Table 4:** Detailed descriptions of the lncRNAs and mRNAs in the Proteoglycans in cancer pathway.

Number*	**Protein**	**mRNA-lncRNA**
①	Ankyrin	ANK3 ↔ FAM86DP, MIAT, LOC646719, RP1-60O19.2, AK123617
②	Tiam1	TIAM1 ↔ RP11-147L13.2
③	ROCK	ROCK2 ↔ XLOC_007896, TPRG1-AS1, U73167.7, LDHA
④	Filamin	FLNB ↔ CTA-313A17.5
⑤	FN	FN1 ↔ TPTEP1, BC033241, U73167.7
⑥	α5β1	ITGA5 ↔ RP11-736K20.5
⑦	GPC1	GPC1 ↔ XLOC_010305, XLOC_001415, NOP14-AS1
⑧	Sdc-2	SDC2 ↔ LOC441204
⑨	MAPK	MAPK12 ↔ AL078621.4

* Represents the number in Figure 5D.

**Table 5:** Detailed descriptions of the lncRNAs and mRNAs in the pathway in cancer.

Number*	**Protein**	**mRNA-lncRNA**
①	ECM	LAMA4 ↔ AK123617
②	Bcl-XL	BCL2L1 ↔ TMEM191A
③	Frizzled	FZD9 ↔ XLOC_000090, RP11-666A20.4
④	BMP	BMP2 ↔ RP11-72I8.1
⑤	EGF	EGF ↔ ABCC6P1, AL078621.4

* Represents the number in Figure 5E.

### Identification of the possible roles of lncRNAs based on genomic co-localization relative to protein coding genes

To further explore the possible roles of differentially expressed lncRNAs during the process of dural penetration in chordoma patients, we analyzed their genomic locations and classified them into lincRNA, enhancer-like lncRNA or antisense lncRNA (Figure 4). Among the 2760 differential expressed lncRNAs between no dural penetration and serious dural penetration chordoma patients, 186 were due to lincRNAs, 109 were enhancer-like lncRNAs, and 143 were antisense lncRNAs. In addition, their associated coding genes were significantly differential expressed in various degrees of dural penetration of chordoma samples (Supplementary Table 2). We combined differentially expressed lincRNAs and enhancer-like lncRNAs with their adjacent coding genes located within 300 kb in the genome to analyze the potential functions of these lncRNAs. All of these lncRNAs and associated mRNAs were shown in Supplementary Tables 6–8.

**Figure 6:**
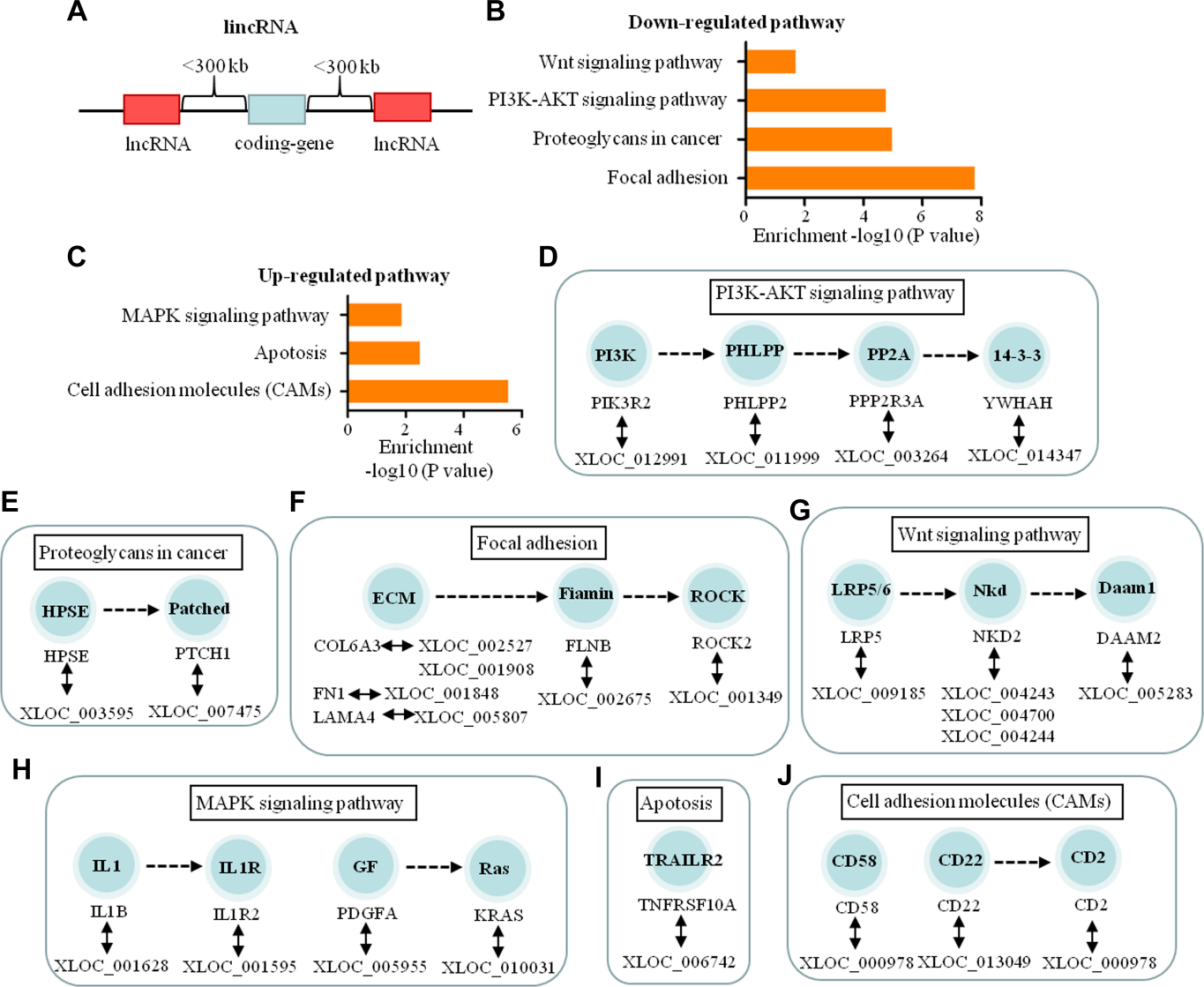
**Functional predictions of the lincRNAs based on pathway analysis of their adjacent coding-genes within 300 kb in the genome.** (**A**) Schematic representation of lincRNA. (**B**, **C**) The pathway analysis applied for 22 mRNAs that were adjacent with 24 lincRNAs and showed the 4 down-regulated and 3 up-regulated pathways related to dural penetration of chordoma (*P* < 0.05). (**D**–**G**) The 17 lincRNAs and their adjacent coding genes are in the down-regulated pathways. (**H**–**J**) The 7 lincRNAs and their adjacent coding genes are in the up-regulated pathways.

LincRNAs are large intergenic non-coding RNA (Figure 6A). The functions of 186 lincRNAs were predicted through pathway analysis of their adjacent genes. The results indicated that 24 lincRNAs may regulate the expression of 22 genes which participate in three up-regulated and four down-regulated significant pathways (Figure 6B, 6C). These pathways regulate tumor cell adhesion, invasion, metastasis and cell apoptosis in the process of dural penetration of chordoma (Figure 6D–6J).

**Figure 7:**
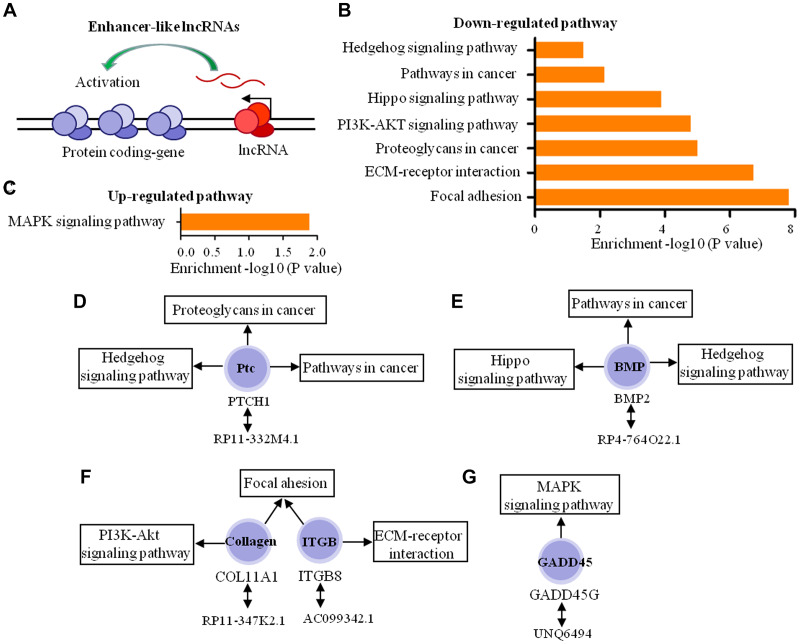
**Biological function predictions of the enhancer-like lncRNAs by pathway analysis of their adjacent coding-genes within 300 kb in the genome.** (**A**) Schematic representation of enhancer-like lncRNA. (**B**, **C**) The pathway analysis applied for 5 mRNAs that regulated by 5 enhancer-like lncRNAs and showed the 7 down-regulated pathways and 1 up-regulated pathways (*P* < 0.05). (**D**–**G**) The 5 enhancer-like lncRNAs, 5 coding genes, and signaling pathways in which these coding genes participated.

We then predicted the roles of enhancer-like lncRNAs (Figure 7A) in the dural penetration of chordoma. Of the 109 enhancer-like lncRNAs, 78 lncRNAs were selected with expression levels positively correlated with coding-gene expression. Therefore, the functions of 78 lncRNAs acting as enhancers were predicted through pathway analysis of 115 coding-genes. The results indicated that five coding-genes participated in seven down-regulated and one up-regulated pathways (Figure 7B, 7C). These pathways represent diverse tumor invasion processes, including cell adhesion, angiogenesis, proliferation and metastasis. These five coding-genes play a key role in tumor progression, and they tend to be involved in more than one signaling pathway. Consequently, we predicted that five enhancer-like lncRNAs, which were RP11-332M4.1, RP11-347K2.1, AC099342, RP4-764O22.1 and UNQ6494, play important roles in dural penetration of chordoma cell (Figure 7D–7G).

**Figure 8:**
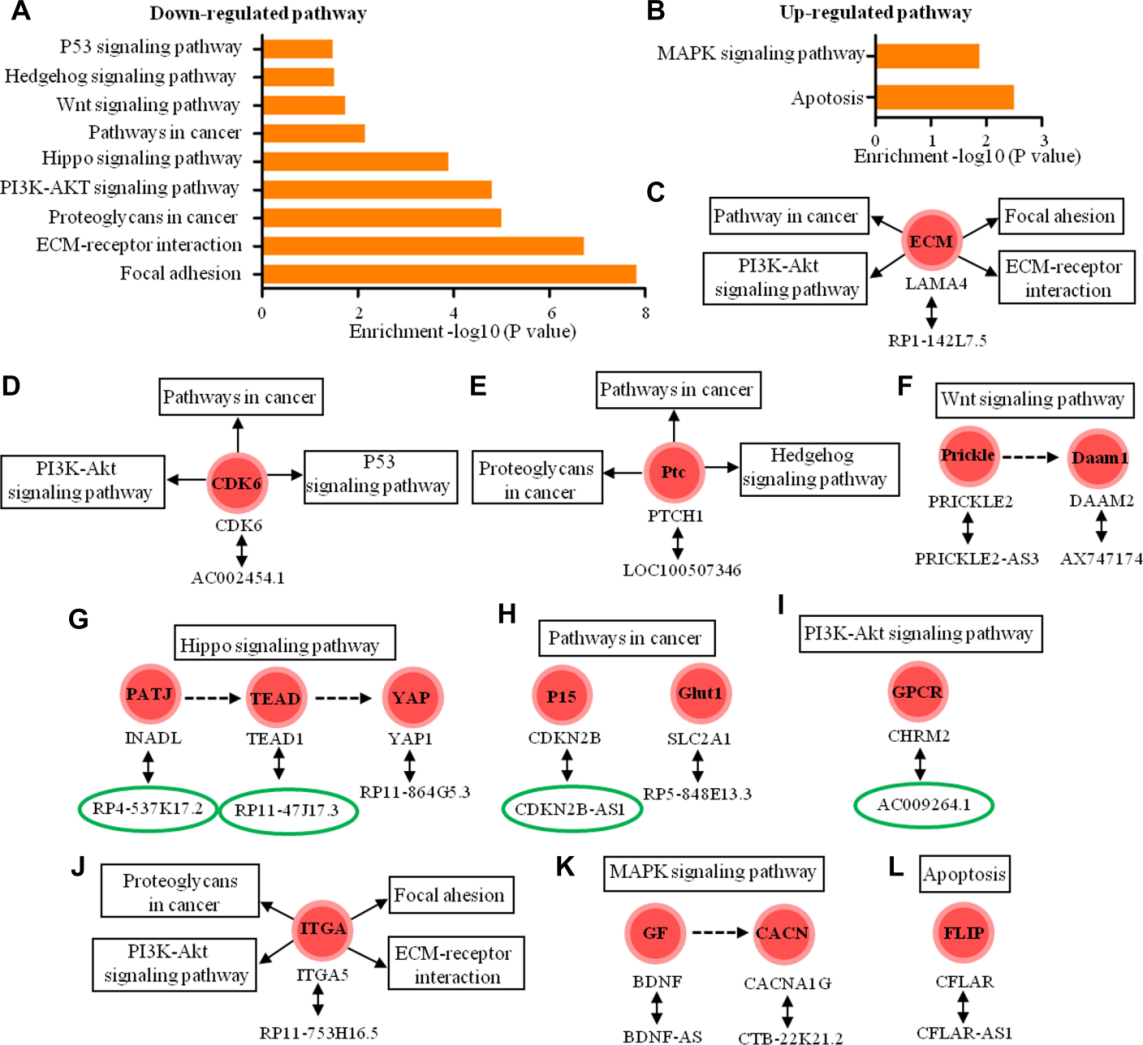
**Functional predictions of the antisense lncRNAs according to pathway analysis of their sense coding genes.** (**A**, **B**) The pathway analysis applied for 15 mRNAs that were sense coding genes of 15 antisense lncRNAs and showed 9 down-regulated pathways and 2 up-regulated pathways (*P* < 0.05) (**C**–**I**) The 11 antisense lncRNAs and their sense coding genes in down-regulated signaling pathways. (**J**–**L**) The 4 antisense lncRNAs and their sense coding genes are in up-regulated signaling pathways. The lncRNAs in the green circle represent intronic antisense lncRNA and other lncRNAs represent natural antisense lncRNA.

Lastly, we analyzed 143 antisense lncRNAs, 63 of which are intronic antisense and 80 of which were natural antisense. Based on KEGG analysis, the biological roles of 15 antisense lncRNAs (four intronic antisense and 11 natural antisense) were predicted, and the results indicated that 14 coding-genes participated in nine down-regulated pathways and two up-regulated pathway (Figure 8A, 8B). These pathways are involved in tight junction, focal adhesion, cell migration and invasion, cell motility and proliferation and apoptosis (Figure 8C–8L).

### qRT-PCR analysis of the expression of lncRNAs and mRNAs in chordoma samples

**Figure 9:**
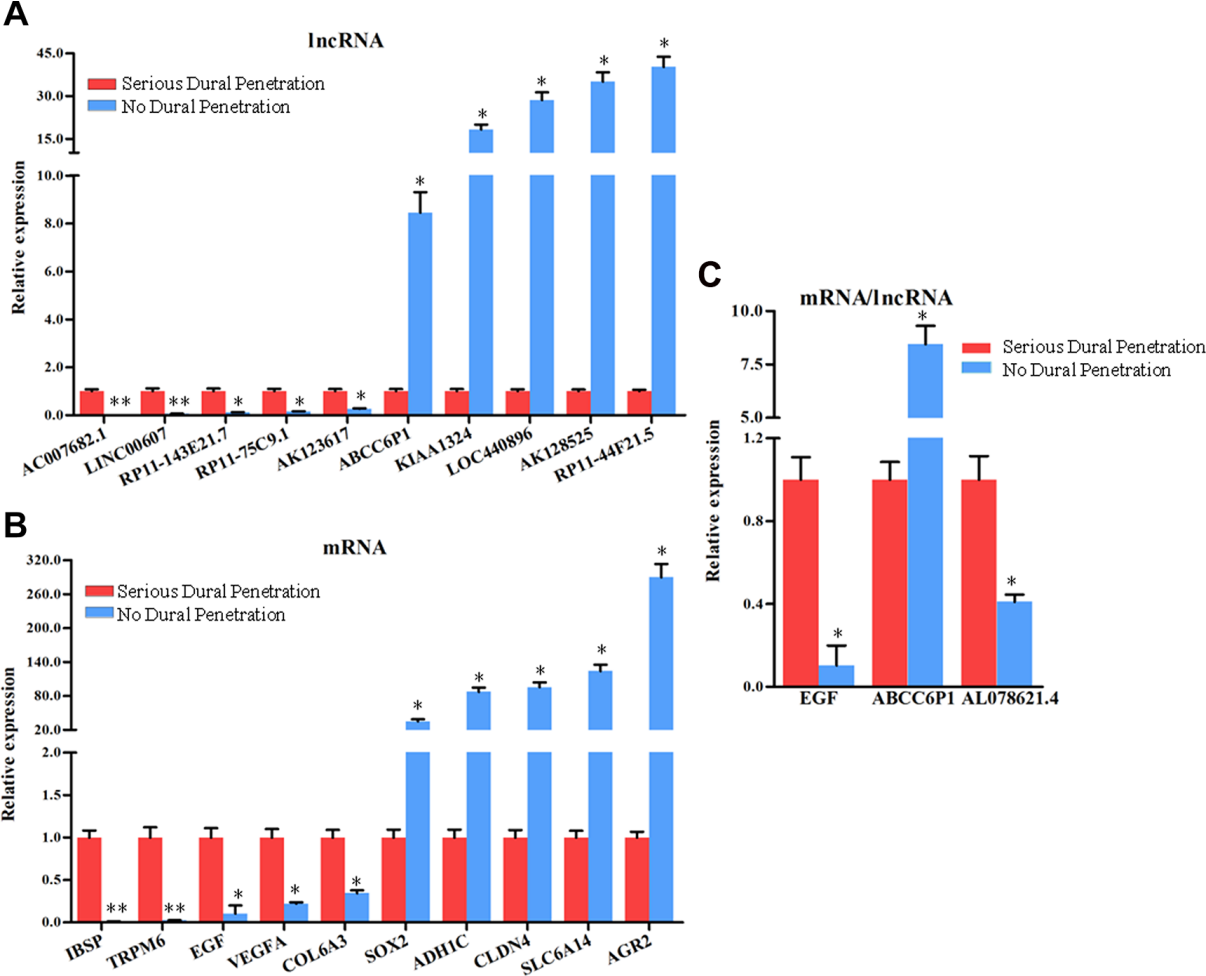
**The expression analysis of the lncRNAs and mRNAs in chordoma tissues with serious dural penetration and without dural penetration.** The expression level of (**A**) 5 down-regulated lncRNAs and 5 up-regulated lncRNAs, (**B**) 5 down-regulated mRNAs and 5 up-regulated mRNAs, (**C**) EGF mRNA and ABCC6P1, AL078621.4 lncRNAs were analyzed using qRT-PCR, with the *GAPDH* gene as an internal control. Error bars represent the standard errors of independent samples.*P<0.05.

To validate the microarray analysis results, we randomly selected 10 lncRNAs and mRNAs with larger fold changes including up-regulated and down-regulated expression from the microarray results and analyzed their expression levels by qRT-PCR in all chordoma tissues, 10 samples with serious dural penetration, and 10 samples without dural penetration. The results showed that the changes were reasonably consistent between microarray and qPCR data, and further confirmed the findings of the lncRNA and mRNA microarray data (Figure 9A, 9B).

Next, we analyzed the expression of EGF mRNA and the lncRNAs ABCC6P1 and AL078621.4 located in the lncRNA-mRNA co-expression network to explore whether EGF was regulated by these differentially expressed lncRNAs in dural penetration chordoma. EGF plays an important role in chordoma. As shown in Figure 9C, the EGF mRNA and lncRNA AL078621.4 were down-regulated, while lncRNA ABCC6P1 was up-regulated in no dural penetration chordoma samples. In the lncRNA-mRNA co-expression network, the expression of ABCC6P1 was strong negatively correlated with EGF (r = -0.9988), and AL078621.4 was strong positively correlated with EGF (r = 0.9981) (Supplementary Table 4).

## DISCUSSION

Our previous study showed that clival chordoma patients with dural penetration prone to have worse outcomes [9]. The aggressive intracranial growth, which often involves vital structures and repeated local recurrence, is the major causes of death in chordoma patients with serious dural penetration. Therefore, identification of novel efficient methods that can inhibit the invasion and recurrence of this type of chordoma are urgently required. In this study, we investigated the lncRNA and mRNA signatures in clival chordoma with dural penetration. We leveraged two strategies for the functional prediction of the differentially expressed lncRNAs: one utilized the lncRNA-mRNA co-expression network [20, 21], while the other classified lncRNAs as lincRNA, enhancer lncRNA or antisense lncRNA and identified the genomic adjacency of differentially expressed mRNAs. To the best of our knowledge, this is the first report to reveal a molecular mechanism underpinning dural penetration in clival chordoma.

We identified a set of 2760 lncRNAs and 3988 mRNAs with differential expression between no dural penetration and serious dural penetration samples. This indicates that RNA homeostasis might be essential for the invasion of chordoma, and their disruption might lead to dural penetration. We found that 1773 out of the 2760 (64.2%) lncRNAs were down regulated, suggesting that decreased lncRNAs play a more important role than increased lncRNAs in dural penetration.

LncRNA-mRNA expression combined with KEGG pathway analysis showed that 55 altered lncRNAs related to migration and invasion, cell apoptosis and proliferation and participated in 5 down-regulated pathways. The pathways proteoglycans in cancer, focal adhesion, and ECM-receptor interaction are major migratory and invasive pathways. The pathway in cancer modulates important biological processes, including evading apoptosis and proliferation, and the PI3K-AKT signaling plays a crucial role in the cell cycle regulation. All of these pathways showed decreased expression in chordoma without dural penetration. The result illustrated that chordoma with serious dural penetration has strong invasion, proliferation, and decreased apoptosis ability.

We also observed differential expression of 24 lincRNAs, 7 enhancer-like lncRNAs, and 14 antisense RNAs modulating dural penetration in chordomas by altering various pathological processes, including cell adhesion, metastasis, invasion, and apoptosis. In addition to the above 5 signaling pathways, 45 of these lncRNAs also participate in the regulation of 4 other down-regulated signaling pathways, including Wnt signaling, Hedgehog signaling, hippo signaling, P53 signaling, and 3 up-regulated pathways, including cell adhesion molecules (CAMs), apoptosis, and MAPK signaling.

Previous reports of receptor tyrosine kinase (RTK) expression in chordomas suggest that these tumors may respond to kinase inhibitor therapy [22]. Chordomas commonly express RTKs and activated signal transduction molecules. Immunohistochemical (IHC) studies have shown that RTKs, such as platelet-derived growth factor receptor (PDGFR), epidermal growth factor receptor (EGFR), MET and HER2, are expressed in chordoma [22]. The EGFR protein is involved in signal transduction through RTKs. AKT is indicative of tyrosine kinase activity. EGFR signaling and Akt were activated in chordomas. Nevertheless, accounts of partial treatment response of metastatic chordoma to combination cetuximab/gefitinib provide further evidence that drugs targeting the EGFR signaling pathway may benefit chordoma patients [8, 23]. We observed that EGFR (fold change: 2.652), MET (fold change: 4.676), AKT3 (fold change: 3.703) and EGF (fold change: 11.094) which is a ligand of EGFR, were down regulated in chordomas without dural penetration. Several lncRNAs including LOC339529; XLOC_010305, NOP14-AS1, RP11-72I8.1, and KRT18P55 were shown to regulate AKT3, and other lncRNAs ABCC6P1 and AL078621.4 were also shown to modulate EGF. Chordoma with serious dural penetration highly expressed EGF and AKT3 and show evidence of EGFR activation suggesting that RTK inhibitors may be useful for the treatment of dural invasion in chordoma.

Additionally, mRNA for EGF and its regulatory lncRNAs levels of ABCC6P1 and AL078621.4 in chordoma tissues with serious dural penetration or without dural penetration were detected by RT-qPCR. Therefore, together with the data presented herein, these findings strongly suggest that EGF was activated during the process of dural penetration in chordoma. Further studies are warranted to determine the role of EGF and these lncRNA in dural penetration of chordoma.

The main limitation of our present study is the small sample size due to the extreme low incidence of clival chordomas. Although we used rigorous inclusion criteria in this exploratory study, further studies with more chordoma samples are still needed to validate this work.

In conclusion, our study found a multitude of differential expression of lncRNAs and mRNAs between chordomas with serious dural penetration and those with no dural penetration, and explored the potential roles of these lncRNAs associated with dural penetration of chordoma. Our data suggested that these dysregulated lncRNAs could serve as useful therapeutic targets and prognostic biomarkers of chordoma with dural penetration.

## MATERIALS AND METHODS

### Patients and specimens

The chordoma samples included in this study were collected from 20 patients with clival chordomas who underwent endoscopic endonasal transsphenoidal surgery at Beijing Tiantan Hospital, among which 10 cases had serious dural penetration and 10 cases did not have dural penetration. Six samples with serious dural penetration and 6 samples with no dural penetration were used in microarray detection. All of the samples were included in the qRT-PCR validation. No patients received chemotherapy or radiotherapy prior to the surgery. Tumor tissues were collected immediately after resection, snap frozen in liquid nitrogen and stored at -80°C. Histological diagnoses were made on formalin-fixed, paraffin-embedded hematoxylin and eosin sections by reviewers blinded to the original diagnosis. The use of the patient samples was approved by the Ethics Committee of Beijing Tiantan Hospital. Written informed consent for the study was obtained from all subjects.

### Total RNA isolation and microarray detection

Total RNA was isolated with Trizol reagents (Invitrogen, USA) and RNeasy Mini Kit (Qiagen, Hilden, Germany). The purity and quantity of RNA were measured using Nanodrop 2000c (Thermo Scientific, USA). Each sample was amplified and transcribed into fluorescent cRNA (complementary RNA) (Arraystar Flash RNA Labeling Kit, Arraystar). The labeled cRNAs were purified by RNeasy Mini Kit (Qiagen). One μg of labeled cRNAs were fragmented and hybridized with the slide of Human LncRNA V3.0 Array (Arraystar). The slides were incubated for 17 hours at 65°C in an Agilent Hybridization Oven. The hybridized array was washed, fixed and scanned using the Agilent DNA Microarray Scanner.

Based on the NetAffx annotation of the probe sets, the Refseq_NR, Ensemble, Gencode, RNAdb, UCSC known gene and lincRNA Cabili of lncRNAs and the RefSeq, Ensemble, and GenBank annotation of mRNAs, a total of 58,944 distinct probes (corresponding to 30,586 lncRNAs and 26,109 mRNAs) were detected by the Arraystar Human LncRNA Microarray V3.0.

### Microarray data analysis

The acquired array images were analyzed by Agilent Feature Extraction software (version 11.0.1.1, Agilent Technologies). Quantile normalization and subsequent data processing were performed using the GeneSpring GX v12.1 software package (Agilent Technologies). After normalization of the data, lncRNAs and mRNAs that were flagged as Present or Marginal (“All Targets Value”) in at least six out of 12 samples were chosen for further data analysis. Differentially expressed lncRNAs and mRNAs between the two groups were identified through fold-change filtering and two-tailed Student’s t-test. Multiple testing correction was performed by calculating the Benjamini-Hochberg false discovery rate (FDR). Fold-change (FC) ≥ 2 and *P* < 0.05 were considered the criteria for significantly differential expression.

### Kaplan-Meier survival analysis

Clinical information was analyzed using SPSS (version 22.0, IBM Corp. Armonk, NY, USA). The Pearson Chi-Square test was used for sex comparisons, and two-sided Student’s *t*-tests were used for age comparisons and follow-up time comparisons between the two groups. Overall survival and progression-free survival were estimated using Kaplan-Meier survival analysis and a 2-sided log-rank test was used to compare between groups.

### Pathway analysis

Pathway analysis was used to identify the significant pathways of differentially expressed genes according to Kyoto Encyclopedia of Genes and Genomes (KEGG) (http://www.genome.jp/kegg/). Differentially expressed genes were mapped to defined KEGG pathways. Enrichment scoring, which is equal to -log10 (P value), was used to measure pathway significance and specificity. Specifically, a two-sided Fisher’s exact test was used to classify the pathway and we computed P values for the pathway of each of the differential genes, *P* < 0.05 was considered statistically significant.

### Construction of the lncRNA-mRNA co-expression network

Differentially expressed lncRNAs and mRNAs were used to construct co-expression networks. The lncRNA-mRNA networks were built according to the signal intensity of specific expression of mRNA and lncRNA determined in the microarray analysis. For each mRNA-lncRNA pair, Pearson correlation coefficients were calculated and significant correlation pairs (r > 0.998 or < -0.998) were selected to construct the network.

### Quantitative real-time polymerase chain reaction (qRT-PCR) validation

We used qPCR to confirm the expression of lncRNAs and mRNAs in chordoma tissues. First-strand cDNA was synthesized from total RNA (1μg) using the SuperScript III First-Strand Synthesis System (Invitrogen, Carlsbad, California, USA). qRT-PCR was performed on an ABI 7500 Fast system (Applied Biosystems, Foster City, California, USA) using the Power SYBR Green PCR Master Mix (Applied Biosystems). The amplification conditions were 95°C for 10 minutes, then 40 cycles at 95°C for 15 seconds and 60°C for 1 minute. All of the primers used are listed in Supplementary Tables 9, 10. Gene expression was normalized to GAPDH mRNA. Expression was calculated using the 2^−ΔΔCT^ method.

## Supplementary Material

Supplementary Figure 1

Supplementary Table 1

Supplementary Table 2

Supplementary Table 3

Supplementary Table 4

Supplementary Table 5

Supplementary Table 6

Supplementary Table 7

Supplementary Table 8

Supplementary Tables 9, 10
