# Supplementary material for: LncRNA and mRNA expression profiles reveal the potential roles of lncRNA contributing to regulating dural penetration in clival chordoma
Source: Aging (Albany NY). 2020 Jun 13;12(11):10809–26. doi: 10.18632/aging.103294 (PMC7346080; doi:10.18632/aging.103294)
Supplement: Supplementary Figure 1 [file aging-12-103294-s008..pdf]

SUPPLEMENTARY FIGURE

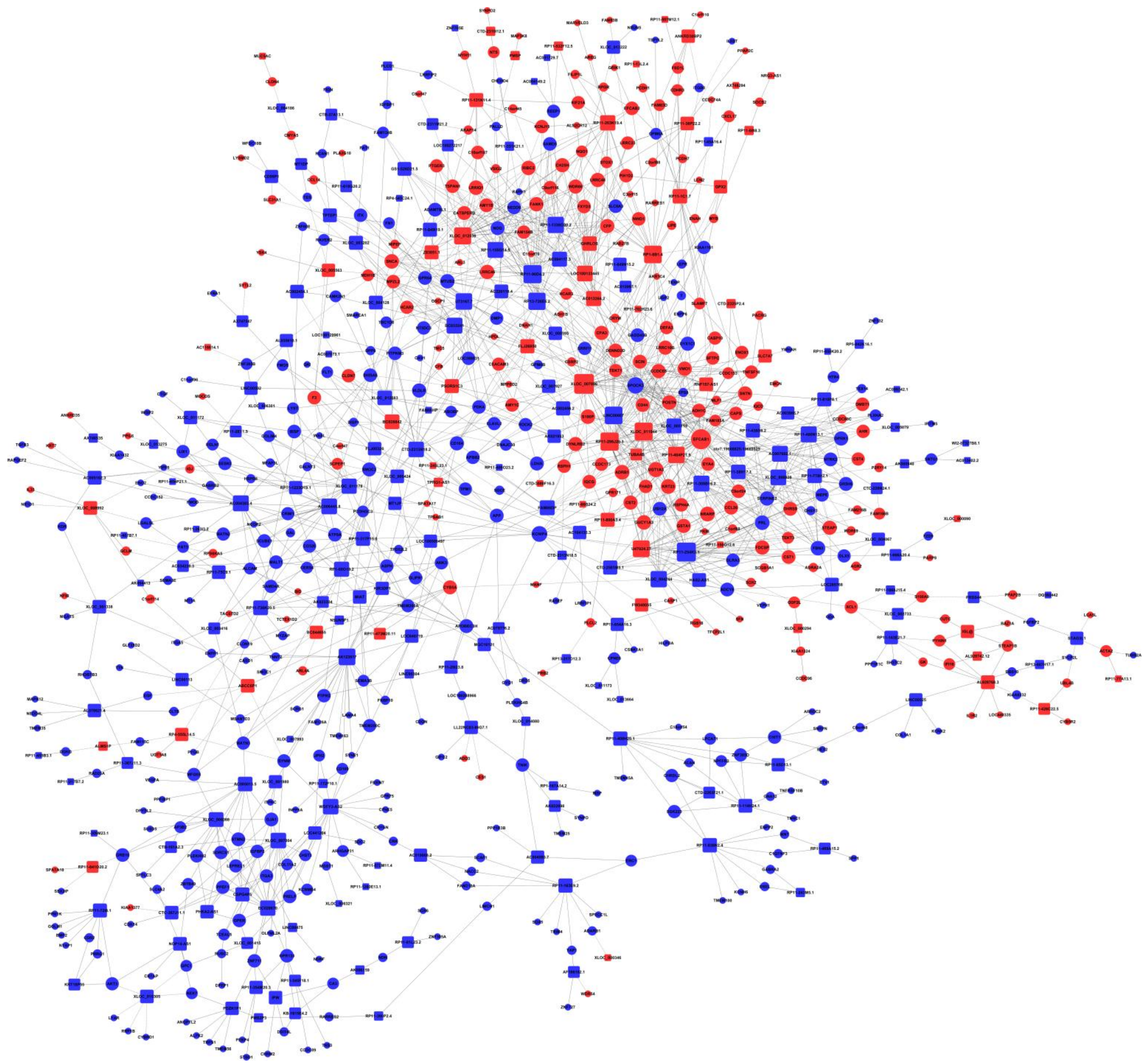

Supplementary Figure 1. LncRNA-mRNA co-expression network in no dural penetration and serious dural penetration chordoma samples. Square: lncRNA, dot: mRNA, red: up-regulated, blue: down-regulated. Solid line represents positive correlation, and dash line represents negative correlation.
