# Supplementary figures and images for: LncRNA and mRNA expression profiles reveal the potential roles of lncRNA contributing to regulating dural penetration in clival chordoma
Source: Aging (Albany NY). 2020 Jun 13;12(11):10809–26. doi: 10.18632/aging.103294 (PMC7346080; doi:10.18632/aging.103294)

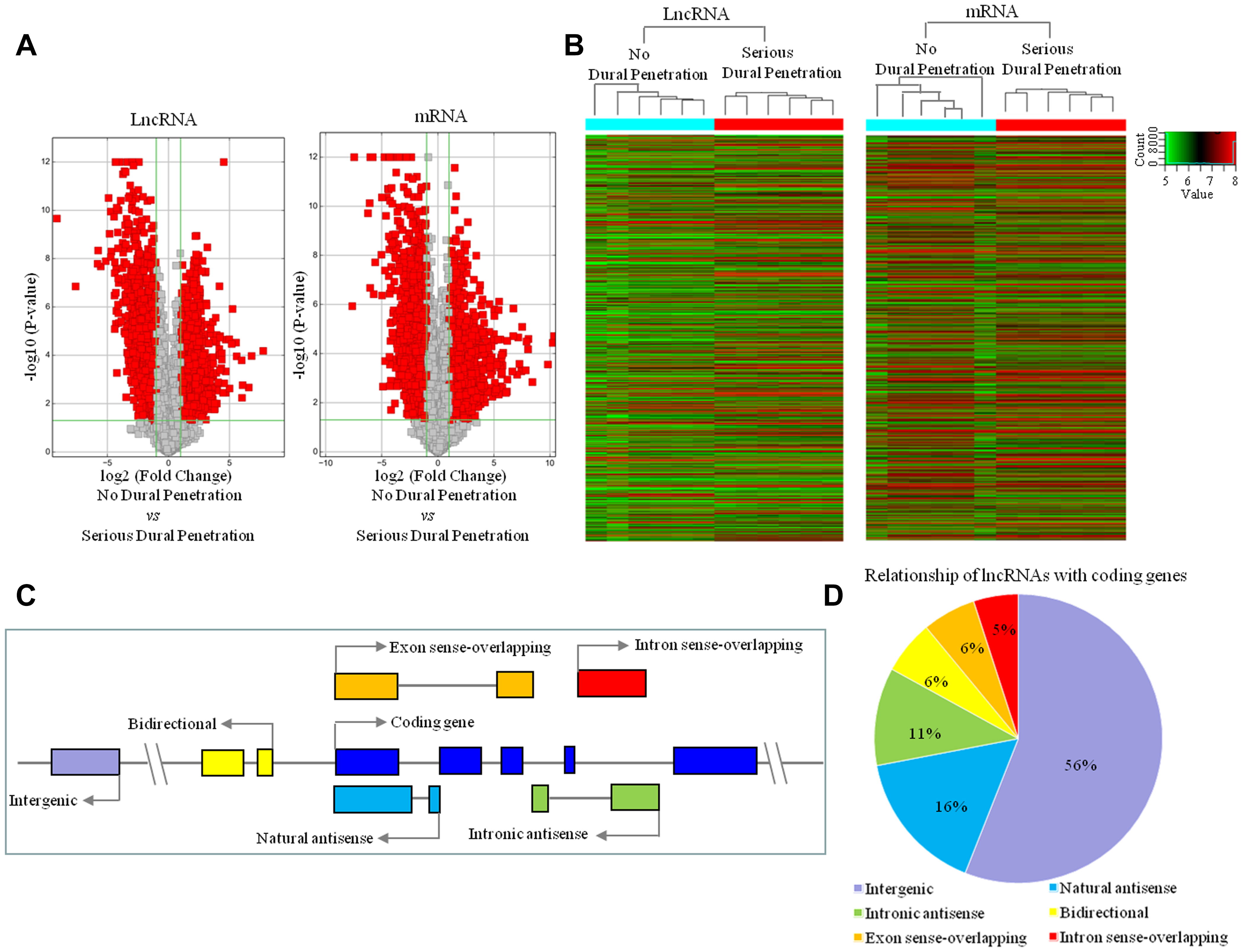

Supplement: Supplementary Table 2 [file aging-12-103294-s010..tif]
