# Supplementary material for: LncRNA and mRNA expression profiles reveal the potential roles of lncRNA contributing to regulating dural penetration in clival chordoma
Source: Aging (Albany NY). 2020 Jun 13;12(11):10809–26. doi: 10.18632/aging.103294 (PMC7346080; doi:10.18632/aging.103294)
Supplement: Supplementary Tables 9, 10 [file aging-12-103294-s007..pdf]

## SUPPLEMENTARY TABLES

**Supplementary Table 9. Primers of lncRNA for qRT-PCR.**

| lncRNA name | Gene ID | Sequence (5'-3') |
|---|---|---|
| AC007682.1 | ENST00000440698 | F: ACTGTCGCTGTTCGGTATTCAC<br>R: TCTCATCATGGCTGCCTATCGT |
| LINC00607 | ENST00000417922 | F: GGGAGGTGGAAGAGAGAAAGCA<br>R: CAGAGGGCATGGATGAGTTGGT |
| RP11-143E21.7 | ENST00000542000 | F: TGTGCTCTGCTGCCCTCATC<br>R: GCATTCAGGGATAGCAAAACGA |
| RP11-75C9.1 | ENST00000430766 | F: GTTTGGAAGCACTGCCTCGTT<br>R: GGCCAAATTACTTATCCTCTCTCGT |
| AK123617 | uc002tji.1 | F: CTTTGCTCAAGGGAGTGTGGAT<br>R: CAGGCTCAGTTTCTCTGGGCT |
| ABCC6P1 | ENST00000565118 | F: GGAACCAGACAGAGCCTGAACC<br>R: TGGTGGTGGATGAAGAGGAGGT |
| KIAA1324 | NR_049773 | F: TGCCAGCCTCTCAGCCAACA<br>R: CGGTGCCAGATTGCTTCAGGTT |
| LOC440896 | uc004afh.2 | F: AGGAGAGCAGGCTTCGGTAGAT<br>R: TCTCGGTCTCTGGAGGACTGTG |
| AK128525 | uc002sti.1 | F: GCCTCATCTCTGGTGCGTCTAT<br>R: GGCTGCTGATGGTGAGAGTGAA |
| RP11-44F21.5 | ENST00000567197 | F: ACCAGCAGAAAGCAGAAGAGC<br>R: AGGCAGTGTAGCATAGTGGGTA |
| AL078621.4 | ENST00000416105 | F: GCCACCACATCCAGCCTACCTA<br>R: AGAGCCTCTCAGAGCAGCATCT |

**Supplementary Table 10. Primers of mRNA for qRT-PCR.**

| mRNA name | Gene ID | Sequence (5'-3') |
|---|---|---|
| IBSP | NM_004967 | F: CACTGGAGCCAATGCAGAAGA<br>R: TGGTGGGGTTGTAGGTTCAAA |
| TRPM6 | ENST00000361255 | F: AGCACAATCATACCCAGCTCA<br>R: CATGGTCTCCAATCAGTCGGC |
| EGF | NM_001178131 | F: GCCTGCTGACACTGAGGATGG<br>R: GCCATCCTCACCAGCCACT |
| VEGFA | NM_001033756 | F: CTGAGTTGCCCAGGAGACCA<br>R: GAGCAGGAAGAGGATGAGGG |
| COL6A3 | ENST00000392003 | F: GTTCCTGGTCCTCATCTCGTC<br>R: TCCTCCTGGTCTGCGTTCCT |
| SOX2 | NM_003106 | F: GCCGAGTGGAAACTTTTGTCG<br>R: GGCAGCGTGTACTTATCCTTCT |
| ADH1C | NM_000669 | F: CTCGCCCCTGGAGAAAGTC<br>R: GGCCCCCAACTCTTTAGCC |
| CLDN4 | NM_001305 | F: TGGGGCTACAGGTAATGGG<br>R: GGTCTGCGAGGTGACAATGTT |
| SLC6A14 | NM_007231 | F: ACCGTGGTAACTGGTCCAAAA<br>R: CGCCTCCACCATTGCTGTAG |
| AGR2 | NM_006408 | F: GTCAGCATTCTTGCTCCTTGT<br>R: GGGTCGAGAGTCCTTTGTGTC |
| GAPDH | NM_002046 | F: GCACCGTCAAGGCTGAGAAC<br>R: TGGTGAAGACGCCAGTGGA |
